# Bacterial and protozoal agents of feline vector-borne diseases in domestic and stray cats from southern Portugal

**DOI:** 10.1186/1756-3305-7-115

**Published:** 2014-03-24

**Authors:** Carla Maia, Cláudia Ramos, Mónica Coimbra, Filipa Bastos, Ângela Martins, Pedro Pinto, Mónica Nunes, Maria Luísa Vieira, Luís Cardoso, Lenea Campino

**Affiliations:** 1 Unidade de Parasitologia Médica, Instituto de Higiene e Medicina Tropical (IHMT), Universidade Nova de Lisboa (UNL), Lisbon, Portugal; 2 Centro de Malária e outras Doenças Tropicais, IHMT-UNL, Lisbon, Portugal; 3 Faculdade de Medicina Veterinária, Universidade Lusófona de Humanidades e Tecnologias, Lisbon, Portugal; 4 Clínica Veterinária Porto Seguro, Olhão, Portugal; 5 Hospital Veterinário da Arrábida, Azeitão, Portugal; 6 Unidade de Microbiologia Médica, IHMT-UNL, Lisbon, Portugal; 7 Centro de Recursos Microbiológicos, Faculdade de Ciências e Tecnologia, UNL, Caparica, Portugal; 8 Department of Veterinary Sciences, School of Agrarian and Veterinary Sciences, University of Trás-os-Montes e Alto Douro, Vila Real, Portugal; 9 Parasite Disease Group, Instituto de Biologia Molecular e Celular, Universidade do Porto, Oporto, Portugal; 10 Departamento de Ciências Biomédicas e Medicina, Universidade do Algarve, Faro, Portugal

**Keywords:** Cats, Feline vector-borne diseases, Bacteria, Protozoa, Portugal

## Abstract

**Background:**

Feline vector-borne diseases (FVBD) have emerged in recent years, showing a wider geographic distribution and increased global prevalence. In addition to their veterinary importance, domestic cats play a central role in the transmission cycles of some FVBD agents by acting as reservoirs and sentinels, a circumstance that requires a One Health approach. The aim of the present work was to molecularly detect feline vector-borne bacteria and protozoa with veterinary and zoonotic importance, and to assess associated risk factors in cats from southern Portugal.

**Methods:**

Six hundred and forty-nine cats (320 domestic and 329 stray), from veterinary medical centres and animal shelters in southern Portugal, were studied. *Anaplasma* spp./*Ehrlichia* spp., *Babesia* spp., *Bartonella* spp., *Borrelia burgdorferi* sensu lato, *Hepatozoon* spp. and *Leishmania* spp. infections were evaluated by polymerase chain reaction (PCR) in blood samples.

**Results:**

One hundred and ninety-four (29.9%) cats were PCR-positive to at least one of the tested genera or complex of FVBD agents. Sixty-four (9.9%) cats were positive to *Leishmania* spp., 56 (8.6%) to *Hepatozoon* spp., 43 (6.6%) to *Babesia* spp., 35 (5.4%) to *Anaplasma* spp./*Ehrlichia* spp., 19 (2.9%) to *Bartonella* spp. and 14 (2.2%) to *B. burgdorferi* s.l. Thirty-three (5.1%) cats were positive to two (n = 29) or three (n = 4) genera/complex. *Babesia vogeli*, *Bartonella clarridgeiae*, *Bartonella henselae*, *Ehrlichia canis*, *Hepatozoon felis* and *Leishmania infantum* were identified by DNA sequencing.

**Conclusions:**

The occurrence of FVBD agents in southern Portugal, some of them with zoonotic character, emphasizes the need to alert the veterinary community, owners and public health authorities for the risk of infection. Control measures should be implemented to prevent the infection of cats, other vertebrate hosts and people.

## Background

Vector-borne diseases comprise a group of globally distributed and rapidly spreading illnesses that are caused by a range of pathogens transmitted by arthropods, including ticks, fleas, mosquitoes and phlebotomine sand flies [1-3]. In addition to their veterinary importance, cats and dogs play a central role in the transmission cycles of some agents of vector borne diseases (e.g. anaplasmosis, bartonellosis, borreliosis and leishmaniosis) by acting as reservoirs, amplifying hosts or sentinels, with such circumstances requiring a One Health approach [4,5].

Feline vector borne diseases (FVBD) have emerged in recent years, showing a wider geographic distribution and increased global prevalence. Environmental, demographic and human behavioral factors (e.g. travelling with pets, changes in social and leisure activities), together with the direct impact of climate changes on the abundance, geographical distribution and vectorial capacity of vector arthropods have contributed to the changing epidemiology of these arthropod-borne diseases [2,6].

The detection of FVBD agents can be challenging as some of them occur in healthy cats, and the clinical signs, whenever present, are normally unspecific of those diseases. PCR-based methods applied to vector-borne pathogens are very effective to detect and characterize infecting organisms, for monitoring cure after chemotherapy and to evaluate the role that subclinically-infected cats can play in the transmission of infections [7].

A recent polymerase chain reaction (PCR) study reported positivity to *Anaplasma*/*Ehrlichia*, *Babesia*, *Hepatozoon, Leishmania* and *Rickettsia* in client-owned cats from the north and centre regions of Portugal [8]. Molecular and serological studies on *Leishmania infantum*[9,10], *Anaplasma phagocytophilum, Bartonella* spp. and *Rickettsia conorii*[11] have been performed in domestic and stray cats from southern Portugal. Nevertheless, information about FVBD agents circulating countrywide is still limited, especially in the south, and therefore the aim of the present study was to assess the presence of bacteria and protozoa with veterinary and zoonotic importance in cats from southern of Portugal, and to assess positivity-associated risk factors.

## Methods

### Cats and samples

From January 2012 to August 2013, a total of 649 cats (320 domestic and 329 stray), from veterinary medical centres and animal shelters in southern Portugal, were studied. Cats were from the districts of Lisbon (n = 282) and Setúbal (n = 104), in the region of Lisbon, and from the district of Faro (n = 263), which overlaps the region of the Algarve. In the Lisbon region most of the domestic cats lived in apartments or in semi-detached houses, while cats from the Algarve region lived in rural areas and used to spend most of their time exclusively outdoors.

Out of the stray cats, 294 were collected to be neutered for birth-rate control or to be housed in a shelter for adoption, and 35 were captured and euthanized in the scope of official animal control programs. Domestic cats were randomly included after obtaining the owners informed consent. In the case of stray cats, written consent for enrolment was also obtained from the person in charge of each shelter.

Whole blood samples were collected by cephalic or jugular venipuncture and spotted onto filter paper (Whatman no. 3) for DNA extraction. Samples were dried at room temperature and kept at 4°C until tested. Whenever available, data on gender, breed, living conditions, age, use of acaricides/insecticides, clinical status (presence or absence of clinical signs compatible with a FVBD), and serological status regarding feline immunodeficiency virus (FIV) and feline leukaemia virus (FeLV) infections were registered for each cat (Table 1). Clinical signs compatible with FVBD comprised anorexia, muscular atrophy, dermatological manifestations, exercise intolerance, fever, gastrointestinal alterations, lameness, lymphadenopathy, muscular lethargy, ocular manifestations, pale mucous membranes or weight loss.

**Table 1:** Comparison of prevalence of FVBD pathogens in different groups of cats from southern Portugal

**Variable/category**	**Nº of tested cats (%)**	**N° of positive cats (%)**						
		***Anaplasma*/*Ehrlichia***	***Babesia***	***Bartonella***	***B. burgdorferi *** **s.l.**	***Hepatozoon***	***Leishmania***	**≥ 1 positive PCR**
**Gender**	649							
Female	372 (57.3)	18 (4.8)	31 (8.3)	11 (3.0)	9 (2.4)	32 (8.6)	44 (11.8)	122 (32.8)
Male	277 (42.7)	17 (6.1)	12 (4.3)	8 (2.9)	5 (1.8)	24 (8.7)	20 (7.2)	72 (26.0)
**Breed**	484							
DSH	432 (89.3)	22 (5.1)	22 (5.1)*	10 (2.3)	4 (0.9)	31 (7.2)	33 (7.6)	109 (25.2)
Other breed	52 (10.7)	1 (1.9)	10 (19.2)*	2 (3.8)	1 (1.9)	3 (5.8)	7 (13.5)	19 (36.5)
**Lifestyle**	649							
Domestic	320 (49.3)	9 (2.8)*	28 (8.8)*	5 (1.6)	3 (0.9)	24 (7.5)	33 (10.3)	91 (28.4)
Stray	329 (50.7)	26 (7.9)*	15 (4.6)*	14 (4.3)	11 (3.3)	32 (9.7)	31 (9.4)	103 (31.3)
**Age** (months)	462							
[3-11]	129 (27.9)	5 (3.9)	13 (10.1)	4 (3.1)	1 (0.8)	3 (2.3)*	6 (4.7)^a^	27 (20.9)
[12-59]	216 (46.8)	10 (4.6)	13 (6.0)	6 (2.8)	0 (0.0)	17 (7.9)	14 (6.5)^b^	54 (25.0)
[60-228]	117 (25.3)	5 (4.3)	5 (4.3)	0 (0.0)	2 (1.7)	11 (9.4)*	16 (13.7)^a,b^	35 (29.9)
**Acaricides-insecticides**	568							
Yes	204 (35.9)	5 (2.5)	4 (2.0)*	3 (1.5)	3 (1.5)	11 (5.4)*	27 (13.2)*	51 (25.0)*
No	364 (64.1)	22 (6.0)	37 (10.2)*	16 (4.4)	10 (2.7)	42 (11.5)*	28 (7.7)*	126 (34.6)*
**Region**	649							
Lisbon	386 (59.5)	18 (4.7)	9 (2.3)*	8 (2.1)	3 (0.8)*	25 (6.5)*	31 (8.0)	85 (22.0)*
Algarve	263 (40.5)	17 (6.5)	34 (12.9)*	11 (4.2)	11 (4.2)*	31 (11.8)*	33 (12.5)	109 (41.4)*
**Habitat**	649							
Urban	282 (43.5)	18 (6.4)	8 (2.8)*	4 (1.4)	0 (0.0)	14 (5.0)*	9 (3.2)*	47 (16.7)*
Rural	367 (56.5)	17 (4.6)	35 (9.5)*	15 (4.1)	14 (3.8)	42 (11.4)*	55 (15.0)*	147 (40.1)*
**Housing**	589							
Totally indoors	124 (21.1)	3 (2.4)	6 (4.8)	1 (0.8)	0 (0.0)	3 (2.4)*	13 (10.5)	24 (19.4)*
Access to outdoors	465 (78.9)	28 (6.0)	36 (7.7)	17 (3.7)	8 (1.7)	51 (11.0)*	39 (8.4)	151 (32.5)*
**FeLV**	242							
Negative	231 (95.5)	12 (5.2)	15 (6.5)	6 (2.6)	6 (2.6)	13 (5.6)	29 (12.6)	70 (30.3)
Positive	11 (4.5)	1 (9.1)	1 (9.1)	0 (0.0)	1 (9.1)	1 (9.1)	0 (0.0)	3 (27.3)
**FIV**	247							
Negative	226 (91.5)	11 (4.9)	16 (7.1)	6 (2.7)	6 (2.7)	14 (6.2)	28 (12.4)	69 (30.5)
Positive	21 (8.5)	3 (14.3)	1 (4.8)	0 (0.0)	1 (4.8)	1 (4.8)	1 (4.8)	6 (28.6)
**Clinical status**	222							
Non-suspect	197 (88.7)	8 (4.1)	26 (13.2)	1 (0.5)	0 (0.0)	12 (6.1)	8 (4.1)	49 (24.9)
Suspect	25 (11.3)	3 (12.0)	1 (4.0)	0 (0.0)	0 (0.0)	1 (4.0)	3 (12.0)	6 (24.0)
**Total**	649 (100)	35 (5.4)	43 (6.6)	19 (2.9)	14 (2.2)	56 (8.6)	64 (9.9)	194 (29.9)

^*,a,b^ Statistically significant difference (*p* <0.05); DSH: domestic short-haired; FeLV: feline leukaemia virus; FIV: feline immunodeficiency virus.

This study was ethically approved by the boards of the Institute of Hygiene and Tropical Medicine (IHMT-UNL) and of the Faculty of Veterinary Medicine (ULHT) as complying with the Portuguese legislation for the protection of animals (Law 92/1995).

### PCR amplification and sequencing

A commercial kit (Kit Citogene®, Citomed) was used to extract DNA from blood on filter paper. Four discs of filter paper (4 mm in diameter each) were incubated with lysis buffer (150 μl) and 1.5 μl of proteinase K (20 mg/ml). Further DNA extraction followed the kit manufacturer’s instructions.

Positivity to *Anaplasma* spp./*Ehrlichia* spp., *Babesia* spp., *Bartonella* spp., *B. burgdorferi* s.l., *Hepatozoon* spp. and *Leishmania* spp. DNA in blood samples was tested by PCR according to previously described protocols (Table 2). PCR amplifications were performed in a 25 μl final volume containing 2 mM MgCl_2_, 1 unit of Taq DNA polymerase (GoTaq DNA Polymerase®, Promega), 10 pmol of each primer (15 pmol in the case of *L. infantum*), 0.2 μM each of dATP, dTTP, dCTP and dGTP (Dntps set®, Bionline, Citomed), and 3 μl of DNA template (5 to 200 ng). In all amplifications a positive control containing genomic target DNA and a negative control without DNA were included. PCR products were visualized under UV illumination after electrophoresis migration on a 1.5% gel agarose stained with 0.2 mg/ml ethidium bromide, using a 100 bp DNA ladder as a marker.

**Table 2:** Primer sets for PCR amplification of FVBD agents

**Pathogen**	**Gene**	**Primers**	**Product size (bp)**	**PCR conditions**	**Reference**
*Anaplasma* spp./*Ehrlichia* spp.	16S rRNA	EHR16SD: 5′-GGT ACC YAC AGA AGA AGTCC-3′	345	95°C, 5 min; 35 cycles [94°C 30 sec, 55°C 30 sec, 72°C 90 sec]; 72°C, 5 min	[12]
		EHR16SR: 5′-TAG CAC TCA TCG TTT ACAGC-3′			
*Babesia* spp.	18S rRNA	PIRO-A: 5′-AAT ACC CAA TCC TGA CACAGG G-3′	400	95°C, 5 min; 35 cycles [94°C 30 sec, 55°C 30 sec, 72°C 90 sec]; 72°C, 5 min	[13]
		PIRO-B: 5′-TTA AAT ACG AAT GCC CCCAAC-3′			
*Bartonella* spp.	16S-23S rRNA	325 s: 5′-CTTCAGATGATGATCCCAAGCCTTCTGGCG-3′	500-800	95°C, 5 min; 55 cycles [95°C 15 sec, 66°C 15 sec, 72°C 15 sec]; 72°C, 1 min	[14]
		1100as: 5′-GAACCGACGACCCCCTGCTTGCAAAGCA-3′			
*B. burgdorferi* s.l.	5S-23S rRNA	Outer primers:	380		[15]
		23SN1: 5′-ACCATAGACTCTTATTACTTTGAC-3′		94.5°C, 1 min; 25 cycles [94°C 30 sec, 52°C 30 sec, 72°C 1 min]; 72°C, 5 min	
		23SC1: 5′-TAAGCTGACTAATACTAATTACCC-3′			
		Inner primers:	225		
		23SN2: 5′-ACCATAGACTCTTATTACTTTGACCA-3′		94.5°C, 1 min; 40 cycles [94°C 30 sec, 55°C 30 sec, 72°C 1 min]; 72°C, 5 min	
		5SCB: 5′-biotin-GAGAGTAGGTTATTGCCAGGG-3′			
*Hepatozoon* spp.	18S rRNA	HEP-F: 5′-ATA CAT GAG CAA AAT CTC AAC-3′	626-666	95 °C, 5 min; 34 cycles [95 °C 20 sec, 55 °C, 30 sec, 72 °C, 90 sec]; 72 °C, 5 min	[16]
		HEP-R: 5′-CTT ATT ATT CCA TGC TGC AG-3′			
*Leishmania* spp.	Small subunit rRNA	Outer primers:	603		[17]
		R221: 5′-GGTTCCTTTCCTGATTTACG-3′		94°C, 5 min; 35 cycles [94°C 30 sec, 60°C 30 sec, 72°C 30 sec]; 72°C, 10 min	
		R332: 5′-GGCCGGTAAAGGCCGAATAG-3′			
		Inner primers:	358		[18]
		R223: 5′-TCCCATCGCAACCTCGGTT-3′		94°C, 5 min; 35 cycles [94°C 30 sec, 65°C 30 sec, 72°C 30 sec]; 72°C, 10 min	
		R333: 5′-AAAGCGGGCGCGGTGCTG-3′			

Twenty per cent of the PCR products (30% in the case of *Bartonella* spp.) were purified with a High Pure PCR Product Purification Kit (Roche® Mannheim) according to the manufacturer’s instructions and directly sequenced (one direction) (Stabvida®), using the same primers as those used for the DNA amplification. Species identity of the obtained sequences was determined according to the closest BLAST match (identity ≥97%) to a GenBank accession and deposited in DNA Data Bank of Japan (DDBJ) (http://www.DDBJ.nig.ac.jp).

### Statistical analysis

Percentages of positivity to FVBD agents relative to the independent variables and categories (Table 1) were compared by the Chi-square or Fisher’s exact tests. A *p* value <0.05 was considered as statistically significant. Analyses were performed with SPSS® 21 software for Windows.

## Results

One hundred and ninety-four (29.9%) cats were PCR-positive to at least one of the tested genera or complex of FVBD agents (Table 2). Sixty-four (9.9%) cats were positive to *Leishmania* spp., 56 (8.6%) to *Hepatozoon* spp., 43 (6.6%) to *Babesia* spp., 35 (5.4%) to *Anaplasma* spp./*Ehrlichia* spp., 19 (2.9%) to *Bartonella* spp. and 14 (2.2%) to *B. burgdorferi* s.l. Thirty-three (5.1%) cats were positive to two (n = 29) or three (n = 4) genera/complex (Table 3).

**Table 3:** **Single and mixed PCR-positivity to genera (*Anaplasma */ *Ehrlichia *, *Babesia*, *Bartonella*, *Hepatozoon *** **and *Leishmania*) and/or complex (*B. burgdorferi *** **s.l.) of FVBD agents in 649 cats from southern Portugal**

**Agent(s)**	**No. of cats (%)**
Single positivity	161 (24.8)
*Anaplasma* spp./*Ehrlichia* spp.	24 (3.7)
*Babesia* spp.	28 (4.3)
*Bartonella* spp.	10 (1.5)
*B. burgdorferi* s.l.	8 (1.2)
*Hepatozoon* spp.	37 (5.7)
*Leishmania* spp.	54 (8.3)
Mixed positivity	33 (5.1)
*Anaplasma* spp./*Ehrlichia* spp. + *Bartonella* spp.	2 (0.3)
*Anaplasma* spp./*Ehrlichia* spp. + *B. burgdorferi* s.l.	1 (0.2)
*Anaplasma* spp./*Ehrlichia* spp. + *Hepatozoon* spp.	2 (0.3)
*Anaplasma* spp./*Ehrlichia* spp. + *Leishmania* spp.	2 (0.3)
*Babesia* spp. + *Bartonella* spp.	3 (0.5)
*Babesia* spp. + *Hepatozoon* spp.	8 (1.2)
*Babesia* spp. + *Leishmania* spp.	2 (0.3)
*Bartonella* spp. + *B. burgdorferi* s.l.	1 (0.2)
*Bartonella* spp. + *Hepatozoon* spp.	2 (0.3)
*B. burgdorferi* s.l. + *Hepatozoon* spp.	1 (0.2)
*B. burgdorferi* s.l. + *Leishmania* spp.	3 (0.5)
*Hepatozoon* spp. + *Leishmania* spp.	2 (0.3)
*Anaplasma* spp./*Ehrlichia* spp. + *Babesia* spp. + *Hepatozoon* spp.	2 (0.3)
*Anaplasma* spp./*Ehrlichia* spp. + *Bartonella* spp. + *Hepatozoon* spp.	1 (0.2)
*Anaplasma* spp./*Ehrlichia* spp. + *Hepatozoon* spp. + *Leishmania* spp.	1 (0.2)
Total	194 (29.9)

As shown in Table 1, the non-use of acaricides/insecticides and living in rural areas were associated with *Babesia* spp. and *Hepatozoon* spp.. Furthermore, the prevalence of *Babesia* spp. and *Anaplasma* spp/*Ehrlichia* spp. was statistically higher in domestic and stray cats, respectively. Cats from exotic breeds (including their crosses) had higher positivity to *Babesia* spp. than domestic short-haired cats. Prevalence of *Hepatozoon* spp. was higher in cats with access to outdoors and in cats older than 60 months (5 years). *Leishmania* spp. was more prevalent in cats aged 12-59 months and in cats aged 60-228 months than in cats younger than 12 months, in cats living in rural habitats and in those protected against ectoparasites. Positivity to *Babesia* spp., to *Hepatozoon* spp. and to *Leishmania* spp. was higher in cats living in the Algarve region. Statistically significant differences were also found for PCR positivity to at least one of the studied agents in cats living in the Algarve region, in cats from rural areas, in cats with access to outdoors and in cats without protection against ectoparasites.

Sequencing confirmed *Hepatozoon felis* in 13 cats [DDBJ accession numbers: AB872992 to AB872995; AB896686 to AB896694], *Babesia vogeli* in eight [DDBJ accession numbers: AB896788 to AB896795], *Leishmania infantum* in five cats [DDBJ accession numbers: AB896681 to AB896685], *Bartonella clarridgeiae* in four [DDBJ accession numbers: AB896695 to AB896698], *Bartonella henselae* in two [DDBJ accession numbers: AB872991 and AB896699] and *Ehrlichia canis* in one cat [DDBJ accession number: AB896787]. Although sequencing results were not obtained for all the products of PCR positive reactions, mainly due to small quantities of amplified DNA, all the obtained sequences revealed an agent species consistent with the PCR result.

## Discussion

The present study represents the first survey on FVBD agents performed in cats from southern Portugal. The overall prevalence of *Leishmania* spp. infection in the present study (9.9%) was higher than the one obtained in domestic cats from the north and centre of the country (0.3%) [8], but lower than the prevalence obtained in domestic (20.3%) and stray (30.4%) cats from Lisbon [9,10], suggesting that the rate of *Leishmania* infection might be dynamic over time, depending on the abundance and distribution of proven vector species in conjunction with the number of infected vertebrate hosts. The significant differences of *Leishmania* spp. prevalence between juvenile and adult or old cats corroborated the results obtained in cats from the north of the country [19] and match the situation previously found in a national serosurvey of *Leishmania* canine infection [20]. Seropositivity to *L. infantum* was significantly higher in dogs and cats older than 2 years of age [19,20], which may probably be explained by a cumulative exposure of older animals to the protozoan parasite. The increased contact with the vectors might also be the reason for a significantly higher prevalence of *Leishmania* infection in the surveyed cats living in a rural environment [20].

*L. infantum* has been reported in cats co-infected with immunosuppressive viruses [21]. However, in this study only one cat was co-infected with FIV, corroborating other studies where no association was observed between the presence of *Leishmania* and of FeLV or FIV infections [10].

The use of topical insecticides on dogs has been shown to be effective in reducing the incidence of canine and human visceral infections. However, in the present work cats treated with acaricides/insecticides presented a higher prevalence of positivity to *Leishmania*. Although the compliance of ectoparasiticide application was not evaluated, this result is not entirely surprising because, even if owners had regularly administered insecticides/acaricides, the only repellents effective against sand flies, the pyrethroids, are toxic to cats. A trend to consider cats as a domestic reservoir of *L. infantum* exists, as infection in domestic and stray cats has been increasingly reported in endemic areas [22]. The potential role of cats in zoonotic leishmaniosis, together with the inexistence of suitable repellents that can be used on cats against sand flies, is a critical issue that should be addressed to prevent feline *Leishmania* infection [22].

The detection of *Hepatozoon* spp. and *H. felis* in cats from southern Portugal reported in the present study, together with the sequenced genetic variants of *H. felis* from cats living in the north and centre [8] suggest that the protozoan is widespread throughout the country. The vectors and route of infection of *H. felis* remain unknown [23], although it was recently amplified from *Rhipicephalus sanguineus* collected from cats and dogs living in the centre and south of Portugal [24].

Sporadic cases of *Babesia canis* and the *Babesia microti*-like piroplasm (syn. *Theileria annae*) infections were reported in three immunocompromised domestic cats from Portugal [25,26]. The overall prevalence of *Babesia* infection (6.9%) obtained in the present study was similar to the 9.1% obtained by Vilhena *et al*. [8], corroborating that piroplasmid infections in cats are frequent and that *B. vogeli* is probably the most common species circulating in felines in Portugal. Cats from the Algarve region, those living in rural habitats or not treated with acaricides/insecticides had a significantly higher prevalence of *Babesia* spp. infection in comparison with cats living in the Lisbon region or in urban areas or chemically protected against ectoparasites, probably due to a higher exposition of the former to the vectors. Differences in the genetic/immune background could be the reason why exotic breeds (including their crosses) presented a higher predisposition of positivity to piroplasms. As the clinical importance of infection with most *Babesia* species in cats remains unknown, as well as the vectors responsible for their transmission [26], further studies are needed to understand the epidemiological relevance of piroplasm infection in the feline population.

Several pathogens belonging to the genera *Anaplasma* and *Ehrlichia* are shared by man and companion animals [1], and there is serological and molecular evidence that cats can be infected with species of these intracellular bacteria [7,21,27]. In fact, antibodies to *A. phagocytophilum* and *E. canis* and DNA of *Anaplasma*/*Ehrlichia* were previously detected in cats from Portugal [8,11,28]. Nonetheless, and to the best of our knowledge, this is the first time in the country that *E. canis* has been molecularly confirmed to infect cats.

The prevalence of positivity to *Anaplasma*/*Ehrlichia* in this work (5.4%) was higher than the 1.0% obtained in Spain [27] and than the 0.6% obtained in cats from the centre and north of Portugal [8]. These differences can be related to the targeted population, as only client-owned cats were evaluated in the two above-mentioned studies. In fact, the prevalence of *Anaplasma*/*Ehrlichia* infection was significantly higher in stray cats in the present study. On the other hand, the seroprevalence of *Ehrlichia* infection in stray cats from the Madrid region was lower than in owned cats [21], thus highlighting that other factors favoring vector-host interactions, such as vector density and geographic distribution, and host immunological status, might play a role in the prevalence of feline ehrlichiosis. Interestingly, our results in combination with those from Vilhena *et al*. [8] seem to follow the trend of significantly higher prevalences of antibodies to *Anaplasma* spp. and *E. canis* in dogs from southern Portugal than in dogs from the northern and central regions of the country [3].

Subclinical infection with *B. clarridgeiae* or *B. henselae*, agents of the cat scratch disease, is frequently reported in cats, which are therefore regarded as a major reservoir for human infection [27,29,30]. Recognised risk factors for bacteraemia in cats are young age (<12 months), infestation with fleas, an outdoor lifestyle and a multicat environment [11,29,30]. Data obtained in the present study corroborates these findings, as most of the cats PCR-positive to *Bartonella* spp. were stray cats and/or with access to outdoors and were not protected against ectoparasites. We report the first molecular evidence of *B. clarridgeiae* infection in cats from Portugal. So far, *B. clarridgeiae* had only been previously detected in *Ctenocephalides felis* fleas from Lisbon and Évora districts [11]. The prevalence of *Bartonella* spp. obtained in the present study (2.9%) was higher than the prevalence (0.3%) described in cats from Madrid, Spain [21], but considerably lower than the one previously reported in cats from Portugal (67.6%) [11]. Prevalence of *B. henselae* (0.3%) was also much lower than the ones previously obtained in Portugal (8.1%) [11] and in Barcelona, Spain (17.5%) [27], while prevalence of *B. clarridgiae* infection (0.6%) was similar to a study conducted in Barcelona (1.0%) [7]. Differences in prevalence could be due to climatic and environmental differences among study areas, which result in more frequent flea infestation or a higher level of *Bartonella* spp. infection among both cats and fleas [21,27].

Borreliosis (or Lyme disease) due to the spirochete *B. burgdorferi* continues to receive intense attention in the milieu of companion animals. Domestic cats are exposed to *B. burgdorferi*, with reported seroprevalence rates of 47-71% in cats from endemic areas of the northeastern USA [31]. Regarding Europe, and to the best of our knowledge, only Shaw *et al.*[32] reported *B. burgdorferi* s.l. infection by PCR, in two clinically suspected cats from the United Kingdom. In the present work, *B. burgdorferi* s.l. DNA was amplified from 2.2% of the screened cats, providing the first molecular evidence of naturally occurring *B. burgdorferi* s. l. infection in cats from Portugal. Nevertheless, the situation of *Borrelia* infection transmission and clinical signs in cats remains a subject for further investigation in Portugal.

The contact with arthropod-borne pathogens varies with the season and depends on the activity and abundance of competent vectors. For instance, feline seropositivities to *A. phagocytophilum* and *E. canis* antigens were shown to be higher during autumn, and in May and November, respectively [27]. As most of the blood samples analysed in the present work were collected from October to May, the effect of the different seasons in the prevalence of infection by the different pathogens was not evaluated. A rural habitat, an outdoor housing or access to outdoors, and the non-use of ectoparasiticides were found to be associated with PCR-positivity to one or more genera/complex of FVBD agents, which is related to a higher exposure of cats to arthropod vectors and the agents they might transmit. As documented for dogs, certain organisms (e.g. *B. vogeli, E. canis, H. canis* and *L. infantum*) might be associated with long-term subclinical infections [1] and, in spite of remaining apparently healthy for months or even years, infected cats might serve as reservoirs to other hosts including humans.

Co-infections with different canine vector-borne pathogens are frequent in dogs living in geographic areas where the presence of competent vectors for the different pathogens overlap [1]. In previous entomological surveys made in the south of Portugal, *L. infantum* was amplified in phlebotomine sand flies [33], *Bartonella* was molecularly detected in *C. felis*[11], while *R. sanguineus* specimens were found to harbour *Anaplasma/Ehrlichia*, *Babesia*, *Borrelia* or *Hepatozoon* DNA [24]. Thus, the detection in the present study of 33 cats co-infected with two or three agents/complex of FVBD is not surprising. Nevertheless, it is important to keep in mind that the occurrence of different combinations of vector-borne pathogens, with a possible dysregulation of the immune system, may lead to a severe and non-characteristic clinical outcome which will further complicate the diagnosis, treatment and prognosis.

## Conclusion

In conclusion, the wide spectrum of FVBD agents identified in southern Portugal, some of them of zoonotic concern, reinforces the importance to alert the veterinary community, owners and public health authorities for the risk of transmission of vector-borne pathogens. Therefore, effective prophylactic measures, such as the use of ectoparasiticides against arthropods, and education and awareness, must be put in place, in order to prevent infection and avoid the dissemination of these pathogens among cats and to other vertebrate hosts including human beings.

## Competing interests

The authors declare that they have no competing interests.

## Authors’ contributions

CM planned, designed and supervised the study, and wrote the manuscript; CR, FB and PP collected samples and clinical data, and performed DNA extraction and molecular analyses; AM and MC collected samples and clinical data; MN performed *B. burgdorferi* s.l. nested-PCR; LuC performed data analysis and revised the manuscript; MLV and LeC reviewed the manuscript. All authors read and approved the final manuscript.
